# Human Cytomegalovirus Induced Aberrant Expression of Non-coding RNAs

**DOI:** 10.3389/fmicb.2022.918213

**Published:** 2022-06-13

**Authors:** Zhongjie Yu, Jing Wang, Fulong Nan, Wenyi Shi, Xianjuan Zhang, Shasha Jiang, Bin Wang

**Affiliations:** ^1^Department of Special Medicine, School of Basic Medicine, Qingdao Medical College, Qingdao University, Qingdao, China; ^2^Oral Research Center, Qingdao Municipal Hospital, Qingdao, China; ^3^Department of Pathogenic Biology, School of Basic Medicine, Qingdao Medical College, Qingdao University, Qingdao, China

**Keywords:** HCMV, ncRNAs, cellular events, target therapy, aberrant expression

## Abstract

Human cytomegalovirus (HCMV) is a β-herpesvirus whose genome consists of double stranded linear DNA. HCMV genome can generate non-coding RNAs (ncRNAs) through transcription in its host cells. Besides that, HCMV infection also changes the ncRNAs expression profile of the host cells. ncRNAs play a key role in maintaining the normal physiological activity of cells, and the disorder of ncRNAs expression has numerous adverse effects on cells. However, until now, the relationship between ncRNAs and HCMV-induced adverse effects are not summarized in detail. This review aims to give a systematic summary of the role of HCMV infection in ncRNAs expression while providing insights into the molecular mechanism of unnormal cellular events caused by ncRNAs disorder. ncRNAs disorder induced by HCMV infection is highly associated with cell proliferation, apoptosis, tumorigenesis, and immune regulation, as well as the development of cardiovascular diseases, and the potential role of biomarker. We summarize the studies on HCMV associated ncRNAs disorder and suggest innovative strategies for eliminating the adverse effects caused by HCMV infection.

## Background

Non-coding RNAs (ncRNAs) are transcribed but not translated, thousands of ncRNAs are produced from gene transcription. ncRNAs can be reclassified as microRNA (miRNA), long non-coding RNA (lncRNA), and circular RNA (circRNA) (Hombach and Kretz, 2016; Liu et al., 2020). Most ncRNAs lose the function of encoding proteins (Zhu et al., 2021) but reportedly have other essential biological functions in all biological processes, including regulation of the profiles of mRNA (Zhou B. et al., 2021) and protein (Yu et al., 2021). ncRNAs play key role in cell growth (Chen et al., 2021), differentiation (Fatica and Bozzoni, 2014), polarization (Mohapatra et al., 2021), apoptosis (Zhao et al., 2020), and cellular defense (Tan Gana et al., 2012). Increasing evidence indicates that aberrant expression of ncRNAs results in critical pathogenesis during viral infection, such as HCMV (Shi L. et al., 2020).

**FIGURE 1:**
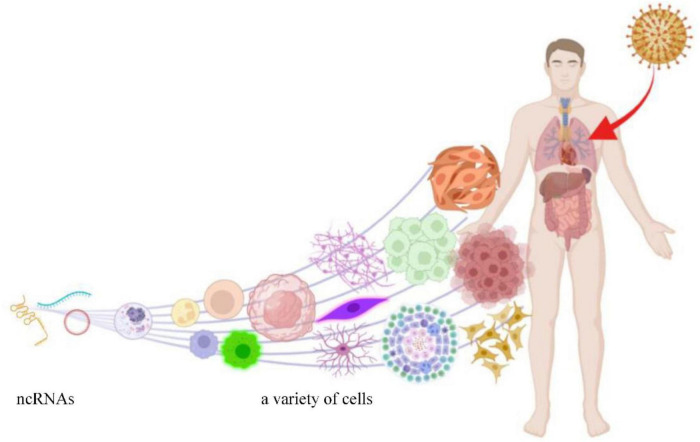
HCMV can infect a variety of cells and induce aberrant expression of ncRNAs (this figure was created in Biorender.com).

HCMV is a kind of DNA virus that consists of double-stranded linear DNA. The genome of HCMV is 236 kb, which encodes 167 genes and translates more than 750 open reading frames (Stern-Ginossar et al., 2012). HCMV can generate its ncRNAs through transcription in the infected cells (Babu et al., 2014), meanwhile changing the expression profile of ncRNAs derived from its host cells (Yan et al., 2019). Most ncRNAs modulated by HCMV benefit viral replication but are harmful to the host. The process of ncRNAs expression regulated by HCMV infection is shown in Figure 1.

The main concerns of most HCMV related reviews focus on the change of immune response or mRNA/proteins. However, the important role of HCMV in the regulation of ncRNAs is neglected habitually. ncRNAs are the key node among HCMV and the altered mRNA. This review summarizes and points out the detailed regulatory role of HCMV in ncRNAs expression and adverse effects on its host cells. Our review’s novel insights will deepen our understanding of the HCMV pathogenic mechanism.

## The Roles of Aberrant Expression of Human Cytomegalovirus-Induced Ncrna in Viral Infection and Cellular Disorder

**FIGURE 2:**
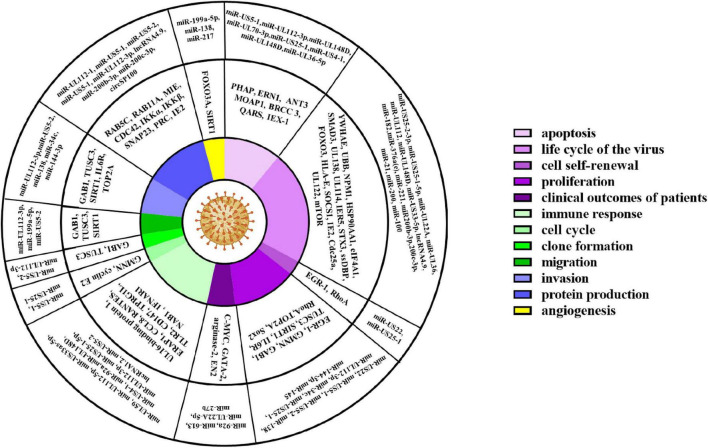
HCMV associated cellular events, and key proteins, ncRNAs.

ncRNAs exert multiple biological properties by regulating the expression level of mRNAs. The homeostasis of ncRNAs is essential to maintain the normal physiological activities of cells, and ncRNAs disorder also results in disastrous consequences for cells, such as the abnormal control of cell cycle and cell death (Liang et al., 2021; Zhou et al., 2022). HCMV is a kind of DNA virus that can produce ncRNAs by transcribing from its genome and interfering with the expression profile of host ncRNAs (Babu et al., 2014; Zhang et al., 2017). These dual changes in the ncRNAs landscape alter the pathophysiology of HCMV infections and accelerate the progression of HCMV related disease. Most aberrant ncRNAs induced by HCMV downregulate the host immune response, apoptosis, and autophagy (Babu et al., 2014). The effects of ncRNAs regulated by HCMV are detailed in Figure 2 and Table 1.

**TABLE 1:** The aberrant expression of non-coding RNAs and cellular events induced by HCMV.

No.	ncRNA	Expression stage	Level	Origin	Regulated protein	Detected sample	Host	Cellular event/Effect	References
1.	miR- UL148D	/	↑	HCMV	PHAP, ERN1	/	/	Counter cellular apoptosis and autophagy	Babu et al., 2014
2.	miR-UL70-3p	/	↑	HCMV	MOAP1	/	/	Counter cellular apoptosis	Babu et al., 2014
3.	miR-US22	/	↑	HCMV	EGR-1	CD34^+^ HPCs	CD34^+^ HPCs	Block self-renewal and proliferation of CD34^+^ HPCs, decrease hematopoietic colony formation	Mikell et al., 2019
4.	miR-US25-1-5p, miR-UL112-3p	6 hpi	↑	HCMV	/	Serum EVs	Infants	Correlate with liver damage	Zhang et al., 2020
5.	miR-US25-1, miR-US25-2-5p and miR-UL112	Latent	↑	HCMV	/	THP-1	THP-1	Disturb melanogenesis, pathways in cancer, endocytosis and wnt signaling pathway	Fu et al., 2014
6.	miR-124-3p	Latent	↑	Human	/	THP-1	THP-1	Disturb melanogenesis, pathways in cancer, endocytosis and wnt signaling pathway	Fu et al., 2014
7.	miR-US25-1-3p	Latent	↑	HCMV	/	Serum	Viremia patients	A predictor for the monitoring of the antiviral treatment of patients suffered with autoimmune diseases	Zhou et al., 2020
8.	miR-US5-2-3p	Frequent reactivation	↑	HCMV	/	Saliva	Renal transplant recipients	Increase T-cell responses to HCMV IE-1 in RTR	Waters et al., 2020
9.	miR-UL22A-5p	/	↑	HCMV	C-MYC	Whole blood	Solid organ transplant patients	Associate with specific virologic and clinical outcomes	Lisboa et al., 2015
10.	miR-UL112-1	24 hpi	↑	HCMV	IL-32	MRC-5	MRC-5	Down-regulate cellular IL-32 transcription and IL-32 protein levels	Huang et al., 2013b
11.	miR-UL59	/	↑	HCMV	Cytomegalovirus UL16-binding protein 1	Plasma	OLP patients	Escape recognition of natural killer cells	Ding et al., 2017
12.	miR-US25-2-3p	/	/	HCMV	eIF4A1	MRC-5	MRC-5	Decrease HCMV and host genomic DNA synthesis, inhibit cap-dependent translation and host cell proliferation	Qi et al., 2013
13.	miR-UL112	/	↑	HCMV	/	HUVECs	HUVECs	Increase the proliferation of HUVECs, raise S-phase fraction	Shen et al., 2018
14.	miR-US5-1	/	↑	HCMV	GMNN	U373	U373	Influence host cell cycle and proliferation	Jiang et al., 2017
15.	mir-US29	Latent	↑	HCMV	/	PBMCs	HCMV IgG positive donors	Maintenance and reactivation of latency	Meshesha et al., 2016
16.	miR-US5-2	Reactivation from latent	↑	HCMV	GAB1	Human fibroblasts	Human fibroblasts	Block the EGF-mediated proliferation of human fibroblasts	Hancock et al., 2020b
17.	miR-UL112-3p	/	↑	HCMV	TUSC3	GBM tissues, cell lines	GBM tissues, cell lines	Promote glioblastoma proliferation, clone formation, migration and invasion	Liang et al., 2017
18.	miR-US25-1-5p	Lytic and latent	↑	HCMV	YWHAE, UBB, NPM1, HSP90AA1	MRC-5	MRC-5	Inhibit viral replication	Jiang et al., 2015
19.	miR-UL112-3p	Late time	↑	HCMV	TLR2	Fibroblasts, monocytic THP1 cells	Fibroblasts, monocytic THP1 cells	Block innate immune response	Landais et al., 2015
20.	miR-214-3p	/	↓	Human	/	Human astrocytoma tissue	Human astrocytoma tissue	Antiviral proprieties	Deshpande et al., 2018
21.	miR-UL-112-3p	/	↑	HCMV	/	Glioblastoma tissue	Glioblastoma tissue	Immune escape, modulate immune microenvironment	Deshpande et al., 2018
22.	miR-UL-70-3p	/	↑	HCMV	/	Human tooth pulps	Human tooth pulps	Dysregulate functions of key host cells that shape oral mucosal immunity, exacerbate disease severity and progression	Naqvi et al., 2018
23.	miR-US5-1, miR-UL112-3p	Lytic	↑	HCMV	IKKα, IKKβ	hAEC, THP-1	hAEC, THP-1	Downregulate proinflammatory cytokine production to create a cellular proviral environment	Hancock et al., 2017
24.	miR-UL112	Immediate-early	↑	HCMV	/	PBMCs	PBMCs	Attenuate NK cell-mediated cytotoxicity	Huang et al., 2015
25.	miR-US5-2	Latent	↑	HCMV	NAB1	HPCs	HPCs	Induce myelosuppression of uninfected CD34^+^ hematopoietic progenitor cells	Hancock et al., 2020a
26.	miR-UL22A	Latent	↑	HCMV	SMAD3	HPCs	HPCs	Maintenance of latency and reactivation	Hancock et al., 2020a
27.	miR-UL36	/	↑	HCMV	UL138	HEK293 cells	HEK293 cells	Contribute to HCMV replication	Huang et al., 2013a
28.	miR-US4-1	/	↑	HCMV	/	Serum	CHB patients	A novel biomarker for predicting the outcome of CHB patients	Pan C. et al., 2016
29.	miR-138	/	↑	Human	SIRT1	MNK-45 cells	MNK-45 cells	Rapid cell growth, enhance invasion capacity	Shi L. et al., 2020
30.	miR-34c	/	↓	Human	IL6R	GC cells	GC cells	Rapid cell growth, enhance invasion capacity	Shi L. et al., 2020
31.	miR-UL112-5p	/	↑	HCMV	ERAP1	GG fibroblasts	GG fibroblasts	Immune evasion	Romania et al., 2017
32.	miR-US25-1	/	↑	HCMV	BRCC 3	EAhy926 cells	EAhy926 cells	Aggravate apoptosis of endothelial EAhy926 cells	Fan et al., 2014
33.	miR-UL112	/	↑	HCMV	UL114	HFF	HFF	Control the life cycle of the virus	Stern-Ginossar et al., 2009
34.	miR-UL148D, miR-US25-1-5p, miR-US5-1	/	↑	HCMV	/	Plasma	Pregnant women with APOs	A potential non-invasive biomarker for predicting and monitoring APOs during HCMV infection	Gao et al., 2021
35.	miR-UL148D	Late stage	↑	HCMV	IER5	Kasumi-3 cells, CD34 + HPCs	Kasumi-3 cells, CD34 + HPCs	Regulate viral latency	Pan C. et al., 2016
36.	miR-UL148D	Later stage	↑	HCMV	RANTES	HFF	HFF	Block immune response	Kim et al., 2012
37.	miR-US4-1	/	/	HCMV	QARS	HELF	HELF	Promote cell apoptosis, benefits the discharge of infectious virus particles	Shao et al., 2016
38.	miR-UL148D	/	↑	HCMV	IEX-1	HEK293	HEK293	Anti-apoptotic effects	Wang et al., 2013
39.	miR-UL36-5p	/	↑	HCMV	ANT3	HEK293, U373, HELF	HEK293, U373, HELF	Inhibit of apoptosis	Guo et al., 2015
40.	miR-US33-5p	/	/	HCMV	STX3	MRC-5	MRC-5	Inhibit HCMV DNA synthesis and of viral replication	Guo et al., 2015
41.	miR-US5-1, miR-UL112-3p	Early time	↑	HCMV	FOXO3a	CD34^+^ HPCs	CD34^+^ HPCs	Protect CD34^+^ HPCs from apoptosis, allow for the establishment of latency and maintenance of viral genome-containing cells	Hancock et al., 2021
42.	miRs UL112-1, US5-1, US5-2	/	↑	HCMV	RAB5C, RAB11A, SNAP23, CDC42	NHDFs	NHDFs	Regulate reorganization of the secretory pathway to control cytokine secretion and facilitate formation of the virion assembly compartment for efficient infectious virus production	Hook et al., 2014
43.	miR-US33as-5p	Lytic and latent	↑	HCMV	IFNAR1	MRC-5, HFF, THP-1	MRC-5, HFF, THP-1	Immune evasion, and achieve lifelong infection	Zhang et al., 2021
44.	miR-UL112-1	/	/	HCMV	IE72	NHDF, U373	NHDF, U373	Decrease in genomic viral DNA levels	Grey et al., 2007
45.	miR-US4-1	/	↑	HCMV	ERAP1	Autologous fibroblasts	Autologous fibroblasts	Cytotoxic T lymphocytes evasion	Kim et al., 2011
46.	miR-US25-1	/	/	HCMV	Cyclin E2	Human primary fibroblast cells, HEK293	Human primary fibroblast cells, HEK293	Cell cycle disorder	Grey et al., 2010
47.	miR-US25-1	/	↑	HCMV	RhoA	CD34^+^ HPCs	CD34^+^ HPCs	Inhibit CD34^+^ HPC self-renewal, proliferation, and hematopoiesis	Diggins et al., 2021
48.	miR-US25-1-5p	Early	↑	HCMV	CD147	U251 MG cells	U251 MG cells	Evade antiviral innate immunity, HCMV inflammatory disorders	Chen et al., 2017
49.	lncRNA4.9	/	↑	HCMV	ssDBP	Human primary foreskin fibroblast cells	Human primary foreskin fibroblast cells	Promote viral DNA replication and viral growth	Tai-Schmiedel et al., 2020
50.	lncRNA4.9	/	↑	HCMV	/	HMECs, breast cancer	HMECs, breast cancer	Contribute to the signaling of oncogenesis	Kumar et al., 2018
51.	lncRNA1.2	/	↑	HCMV	TPRG1L	Human fibroblasts	Human fibroblasts	Impact downstream immune responses	Lau et al., 2020
52.	lncRNA4.9	Latent	↑	HCMV	PRC, MIE	CD14^+^monocytes	CD14^+^ monocytes	Represses transcription	Rossetto et al., 2013
53.	CMV70-3P	/	↑	HCMV	/	Primary glioma cells	Primary glioma cells	Increases GBM CSC stemness, proliferate and form neurospheres	Ulasov et al., 2017
54.	miR-613	/	↓	Human	Arginase-2	Glioblastoma specimens/cells	Glioblastoma specimens/cells	Presence of unfavorable variables, including tumor size, World Health Organization stage, the overall survival and disease-free survival of patient. Anti-apoptosis, promote glioblastoma cell growth, clone formation, invasion and migration	Wang L. et al., 2017
55.	miR-182	/	↑	Human	FOXO3	U-251MG, NPCs cells	U-251MG, NPCs cells	Result in the induction of IFN-I response and suppression of HCMV replication in neural cells	He et al., 2018
56.	miR-376a (e)	/	↑	Human	HLA-E	Human decidual organ	Human decidual organ	Render HCMV infected cells susceptible to elimination by NK cells	Nachmani et al., 2014
57.	miR-200b-3p, 200c-3p	/	↓	Human	IE2	Gastrointestinal tract, bronchi, lungs	Gastrointestinal tract, bronchi, lungs	Associate with cytopathic inflammation due to HCMV infection	Lee et al., 2018
58.	miR-221	/	↑	Human	SOCS1	NPCs	NPCs	Restrain HCMV replication and tissue injury	Yan et al., 2019
59.	miR-144-3p	/	↓	Human	TOP2A	Glioblastoma	Glioblastoma	Inhibit the proliferation, clone formation, and invasion of HCMV-positive glioma	Song et al., 2018
60.	miR-217	24 hpi	↑	Human	FOXO3A	ECs	ECs	Induce angiogenesis	Zhang et al., 2013a
61.	miR-1929-3p	/	↓	Mouse	/	Vascular	Mouse	Increase the blood pressure, promote vascular remodeling, cause endothelial cell injury	Zhou W. et al., 2021
62.	miR200b-3p, 200c-3p	/	↓	Human	IE2	Pretransplant PBMCs	SOT recipients	Control HCMV replication post-transplant	Han et al., 2017
63.	miR-92a	Latent	↓	Human	CCL8	Myeloid cells	Myeloid cells	Lead to evasion of the immune system	Poole et al., 2014
64.	miR-21	/	↓	Human	Cdc25a	NPCs, U-251MG cells	NPCs, U-251MG cells	Increase viral gene expression and production of infectious progeny	Fu et al., 2015
65.	circ_0001445, circ_0001206	Latent	/	Human	/	Whole blood	HCMV-infected patients	Serv as biomarkers of HCMV-infection	Lou et al., 2019
66.	miR-138	/	↑	Human	SIRT1	HUVECs	HUVECs	Enhance endothelial angiogenesis	Zhang et al., 2017
67.	miR-145	/	↓	Human	Sox2	GSCs	Glioblastoma patient	Enhance growth as tumorspheres and intracranial tumor xenografts	Soroceanu et al., 2015
68.	miR-27b	24 hpi	↑	Human	EN2	Human glioma U251 cells	Human glioma U251 cells	Relate to the development of neurological disorders with the HCMV infection	Wang L. et al., 2017
69.	miR-199a-5p	24 hpi	↑	Human	SIRT1	ECs	ECs	Promote cellular migration, tube formation and angiogenesis	Zhang et al., 2013b
70.	miR-200	Latent	/	Human	UL122	Primary CD34^+^ cells	Primary CD34^+^ cells	Maintenance of viral latency	O’Connor et al., 2014
71.	miR-100	/	/	Human	mTOR	MRC-5	MRC-5	Inhibit production of infectious progeny	Wang et al., 2008
72.	miR-92a	Latent	↓	Human	GATA-2	CD34^+^ cells	CD34^+^ cells	Maintenance of latent viral genomes, increased survival	Poole et al., 2011
73.	mmu-miR-1929-3p	/	↓	Mouse	Ednra	Thoracic aorta, heart tissues, peripheral blood	MCMV-infected mice	Raise the blood pressure	Shi Y. et al., 2020
74.	miR-183-5p, miR-210-3p	Congenital	↑	Human	/	Plasma	Infants	Use as disease biomarkers	Kawano et al., 2016
77.	circSP100	/	↑	Human	257 proteins	HELF	HELF	Involve in the spliceosome, protein processing, ribosome, and phagosome pathways	Deng et al., 2021

### The Altered ncRNAs Benefit Human Cytomegalovirus Replication, Latency, and Reactivation

Many ncRNAs are preferentially used in the infected cells to create a cellular viral-friendly environment to ensure the replication, latency, and reactivation of HCMV.

Firstly, raw materials from the host were utilized by HCMV to complete self-replication and product infectious progeny. ncRNAs play a key regulatory role in this process. It was reported that miR-UL36 contributed to HCMV replication by down-regulating UL138 expression (Huang et al., 2013a). Hook et al. (2014) pointed out that HCMV miRs-UL112-1, US5-1, and US5-2 coordinately regulated reorganization of the secretory pathway to control cytokine secretion and facilitate the formation of the VAC for efficient infectious virus production. HCMV-encoded lncRNA4.9 formed an RNA-DNA hybrid (R-loop) through its G + C-rich 5’ end, which played an important role in initiating viral DNA replication (Tai-Schmiedel et al., 2020).

lncRNA beta2.7 is the most highly transcribed viral gene during lytic and latent infection (Gatherer et al., 2011; Stern-Ginossar et al., 2012; Shnayder et al., 2018). This RNA plays an anti-apoptotic role during infection by directly binding with complex I and also through mitigation of reactivate oxygen species (ROS) production (Reeves et al., 2007; Perera et al., 2022). The anti-apoptotic property of lncRNA beta2.7 is essential for successful completion the life cycle of HCMV.

In contrast, some cellular ncRNAs displayed an anti-virus effect by inhibiting HCMV replication. miR-100 and miR-101 modestly suppressed the production of infectious progeny by depressing mTOR expression (Wang et al., 2008). miR-182 suppressed HCMV replication by inducing type I interferon (IFN-I) through the FOXO3/interferon regulatory factor 7 (IRF7) pathway (He et al., 2018). Besides that, miR-200b-3p, miR-200c-3p and miR-21 also could inhibit HCMV replication (Fu et al., 2015; Han et al., 2017; Lee et al., 2018). However, HCMV selectively downregulated the anti-virus cellular miRNAs to help its replication.

Next, ncRNAs exert multiple functions to maintain latent infections. During the late stages of latent HCMV infection, miR-UL148D inhibited immediate early response gene 5 (IER5) expression, thereby rescuing the expression and activity of CDC25B to promote HCMV latency (Pan C. et al., 2016). miR-200 targeted the immediate-early protein 2 (IE2) 3’ untranslated region, resulting in repression of this viral protein to maintain latent infections (O’Connor et al., 2014). Decreased miR-92a contributed to the maintenance of latent viral genomes by increasing the expression of GATA-2 and cellular IL-10 (Poole et al., 2011). In addition, miR-US25-1, miR-UL112-3p, miR-US29, and miR-92a, etc. could play a critical role in maintenance of latency (Mohammad et al., 2014; Poole et al., 2014; Jiang et al., 2015; Meshesha et al., 2016; Diggins et al., 2021).

Furthermore, ncRNAs participated in the regulation of HCMV reactivation. Hancock et al. (2020b) reported that miR-US5-2 directly downregulated epidermal growth factor receptor (EGFR) adaptor protein GAB1, which regulated EGR1 and UL138 expression by affecting downstream MEK/ERK signaling, and then played a key role during reactivation from latency. miR-US25-1-3p significantly upregulated in the reactivation autoimmune patients than others and exhibited an obvious shift-switch from latency to reactivation (Zhou et al., 2020).

Together, these findings provided specific targets for anti-HCMV treatment by regulating replication, latency, and reactivation of HCMV.

### The Altered ncRNAs Interfere Proliferation, Apoptosis and Cell Cycle of Host Cells

Besides the expression of viral ncRNAs in host cells, HCMV infection also changed the profiles of ncRNAs encoded by the host genome. These two sources of ncRNAs are involved in pathways of proliferation, apoptosis, and cell cycle progression, all of which may be implicated in viral pathogenesis.

During HCMV infection, the proliferation of host cells was affected by ncRNAs. It’s reported that viral miR-US22 downregulated EGR-1 and inhibited CD34^+^ HPCs self-renewal and proliferation (Mikell et al., 2019). Conversely, Shen et al. (2018) demonstrated that ectopically expressed miR-UL112 in HUVECs significantly increased proliferation.

ncRNAs also played a key role in regulating cell apoptosis. For example, miR-UL70-3p and UL148D countered cellular apoptosis and autophagy by regulating proapoptotic genes MOAP1, PHAP, and ERN1 (Babu et al., 2014). mir-UL148D exerted anti-apoptotic property by downregulating immediate early gene X-1 (IEX-1) (Wang et al., 2013). miR-UL36-5p inhibited apoptosis by directly downregulating adenine nucleotide translocator 3 (ANT3) (Guo et al., 2015). miR-US5-1 and miR-UL112-3p protected CD34^+^ HPCs from virus-induced apoptosis by mediating FOXO3a/BCL2L11 pathway (Hancock et al., 2021). However, to benefit the discharge of infectious virus particles, miR-US4-1 promotes the apoptosis of the infected cell by silencing the expression of glutaminyl-tRNA Synthetase (QARS) (Shao et al., 2016).

Moreover, ncRNAs encoded by HCMV also affected cell cycle progression. Grey et al. (2010) reported that miR-US25-1 disordered the cell cycle by regulating the expression of cyclin E2, BRCC3, EID1, MAPRE2, and CD147. Ectopically expressed miR-UL112 in HUVECs significantly raised the S-phase fraction in the cell cycle (Shen et al., 2018).

Collectively, these reports demonstrated that ncRNAs mediated signaling pathways, including proliferation, apoptosis, and cell cycle of host cells, played critical roles in the physiological effects of HCMV induced diseases.

### The Effects of ncRNAs in Oncomodulatory

Cancer is the second leading cause of mortality globally, accounting for about 10 million deaths in 2020 (Jameus et al., 2021). Increasing evidence indicates that the products of the HCMV genome are involved in oncomodulatory (Dziurzynski et al., 2012), particularly ncRNAs.

During HCMV latent infection, miR-US25-1, miR-US25-2-5p, miR-UL112, and miR-124-3p were upregulated, and the target genes of those miRNAs were involved in melanogenesis and pathways in cancer (Fu et al., 2014). miR-UL112-3p promoted glioblastoma cell proliferation, clone formation, migration, and invasion by directly regulating tumor suppressor candidate 3 (TUSC3), and the miR-UL112-3p expression was positively associated with glioma size, differentiation, WHO stage and the overall and disease-free survival of patients (Liang et al., 2017). In addition, HCMV lncRNA4.9 gave rise to fast-growing triple-negative tumors in NSG mice (Kumar et al., 2018).

Besides the ncRNAs encoded by its genome, HCMV also employed the ncRNAs encoded by the host genome to promote cancer development. HCMV infection affected the progression of GC by regulating the miR-34c/IL6/STAT3 pathway (Shi L. et al., 2020). miR-27b played a key role in developing HCMV induced neurological disorders by affecting the growth of glioma cells (Wang L. et al., 2017). CMV70-3P miRNA increases glioblastoma multiforme (GBM) cancer stem cells (CSC) stemness (Ulasov et al., 2017). Moreover, HCMV infection contributed to migration and tube formation of endothelial cells through downregulation of SIRT1/eNOS by miR-199a-5p, which contributed to the progress of cancer (Zhang et al., 2013b).

Even worse, HCMV can depress the expression of ncRNAs, which have an anti-cancer function. HCMV upregulated the expression of Sox2 *via* inhibiting miR-145 and subsequently enhanced the stemness and proliferation of GBM cells (Soroceanu et al., 2015). HCMV reduced the level of miR-613, and the reduction of miR-613 expression also correlated to the unfavorable variables of cancer patients (Wang Y. et al., 2017). The expression of miR-144-3p was suppressed by HCMV and then decreased its anti-cancer property (Song et al., 2018).

ncRNAs disorder induced by HCMV infection is highly linked with cancer patients’ poor outcomes. Targeting the changed ncRNAs represented a promising therapeutic strategy for HCMV-related cancers.

### The Effects of ncRNAs in Immune Regulation

During HCMV infection, the host immune system exerts a key antiviral property; however, ncRNAs are employed by HCMV to change the immune microenvironment, escape immunological surveillance and benefit virus survival.

ncRNAs encoded by HCMV contributed to immune evasion by reducing the production of cytokines. It was reported that miR-UL112-1 could functionally down-regulate the level of IL-32 (Huang et al., 2013b), and miR-UL112-3p reduced the expression of multiple cytokines (IL-1β, IL-6, and IL-8) by directly down-regulating TLR2 (Landais et al., 2015). miR-US5-2 suppressed the transcriptional repressor NGFI-A binding protein (NAB1) to induce myelosuppression of uninfected CD34^+^ HPCs by increasing TGF-β secretion (Hancock et al., 2020a). Moreover, HCMV lncRNA1.2 mediated downstream immune responses through manipulating intrinsic NF-κB-dependent cytokine and chemokine release (Lau et al., 2020). miR-US5-1 and miR-UL112-3p limited the production of pro-inflammatory factors (Hancock et al., 2017).

ncRNAs also contributed to the virus-infected cells evading immune cells’ killing. miR-UL59 inhibited the function of natural killer cells by downregulating cytomegalovirus UL16-binding protein 1 (Ding et al., 2017). miR-UL112 blocked NK cell cytotoxicity by suppressing the expression of IFN-1 (Huang et al., 2015). miR-UL148D decreased the aggregation of immune cells by downregulating the chemokine RANTES (Kim et al., 2012). miR-US4-1 and miR-UL112-5p reduced the level of ERAP1, thereby inhibiting the presentation of the HCMV-derived peptides to specific CTLs (Kim et al., 2011; Romania et al., 2017), thus leading to less susceptibility of infected cells to HCMV-specific CTLs. Inversely, edited-miR-376a contributed NK cells to eliminate HCMV infected cells by downregulating the immune-modulating molecule HLA-E (Nachmani et al., 2014). Those results identify a novel immune evasion mechanism mediated by ncRNAs derived from HCMV.

Besides that, miR-US25-1-5p evaded innate antiviral immunity by regulating Cyclophilin A-CD147-ERK/NF-κB pathway targeting CD147 (Chen et al., 2017). miR-US33as-5p downregulated the expression of IFN-stimulated genes (ISGs), inhibited STAT1 translocation into the nucleus, and subsequently evaded the immune system’s killing (Zhang et al., 2021). Moreover, in clinical research, miR-US5-2-3p increased T-cell responses and HCMV reactivation in renal transplant recipients (Waters et al., 2020). In HCMV positive patients, antiviral miRNA, miR-214-3p, was remarkedly decreased with astrocytoma progressing. miR-UL-112-3p was significantly upregulated in glioblastoma and contributed to immune escape of glioblastoma (Deshpande et al., 2018).

miR-221 positively regulates the phosphorylation and activation of NF-κB by directly suppressing the suppressor of cytokine signaling 1 (SOCS1) expression. Moreover, miR-221 alleviates CMV-induced tissue injury by promoting the production of IFN- I and ISGs. Thus, miR-221 can be served as an intrinsic antiviral factor and developed as a treatment target for anti-HCMV treatment (Yan et al., 2019).

Collectively, these findings demonstrated that ncRNAs mediated immune escape by regulating multiple pathways. Targeted these ncRNAs may provide an effective strategy for HCMV treatment.

### ncRNAs Contribute to the Development of Cardiovascular Diseases

Cardiovascular diseases are one of the main causes of morbidity and mortality in developed and developing countries, affecting millions of people yearly (Zuraini et al., 2021). Recently, studies found that HCMV infection is involved in the development of cardiovascular diseases, one of the risk factors is ncRNAs disorder.

These studies revealed that miR-US25-1 accelerated the development and severity of HCMV-induced atherosclerosis by aggravating ox-LDL-promoted apoptosis (Fan et al., 2014). miR-217 and miR-138 promoted HCMV-induced angiogenesis by depressing the expression of SIRT1 (Zhang et al., 2013a,^2017^). In addition, Zhou W. et al. (2021) reported that miR-1929-3p could improve MCMV-induced vascular remodeling and endothelial cell injury, possibly through the deactivation of the NLRP3 inflammasome by ET-1/endothelin A receptor (Ednra). MCMV infection reduced mmu-miR-1929-3p expression, subsequently increased Ednra expression level, and raised the blood pressure (Shi Y. et al., 2020). These findings provide novel insights into HCMV-related cardiovascular diseases.

## Ncrnas Serve as Potential Biomarkers of Human Cytomegalovirus Infection

Accumulating evidence indicated that HCMV is involved in the occurrence and development of numerous diseases. Thus, it’s essential to screen out biomarkers of HCMV infection. With the development of the sequencing technique, a growing number of specific ncRNAs were found in HCMV infections. These ncRNAs not only mediated cellular events induced by HCMV and also could be emerged as potent biomarkers.

It was reported that the serum level of miR-US4-1 could be used to classify chronic hepatitis B (CHB) patients who were and were not responsive to IFN-α treatment with a correct rate of 84.00 and 71.74%, respectively (Pan Y. et al., 2016). Thus, miR-US4-1 could serve as a novel biomarker for predicting the outcome of a CHB patient’s treatment with IFNα. The plasma level of miR-US25-1-5p was significantly increased in pregnant women with adverse pregnancy outcomes (APOs), and the sensitivity and specificity were 68 and 71%, respectively (Gao et al., 2021). This miRNA could be a potential non-invasive biomarker for monitoring APOs during HCMV infection. miR-UL22A-5p is strongly linked with solid organ transplant patients’ specific virologic and clinical outcomes (Lisboa et al., 2015). Levels of miR-US25-1-5p and miR-UL112-3p in serum from infants with HCMV active infection were significantly correlated with liver damage (Zhang et al., 2020). Thus, these ncRNAs could further serve as biomarkers to monitor HCMV related diseases. Moreover, miR-US4-5p and miR-UL112-3p could be selected for cytomegalovirus diagnosis and follow-up (Mohammad et al., 2014; Caputo et al., 2019).

Like the ncRNAs encoded by the HCMV genome, the ncRNAs derived from the host genome could also be denoted as biomarkers. Kawano et al. (2016) revealed that plasma levels of miR-183-5p and miR-210-3p were significantly higher in patients with congenital CMV infection. miR-155 showed a significant difference between kidney transplant patients negative for HCMV infection and positive patients (Bergallo et al., 2018). In addition, the levels of circular RNAs hsa_circ_0001445 and hsa_circ_0001206 were significantly different in HCMV-infected patients vs. normal controls (Lou et al., 2019). These findings suggested that these ncRNAs could potentially serve as biomarkers of HCMV related disease.

## Conclusion and Future Perspectives

ncRNAs exert multiple functions by regulating the expression level of cellular proteins (Deng et al., 2021). HCMV infection changed the expression profiles of ncRNAs, which was the leading cause of HCMV related diseases (Zhang et al., 2016; Mohammad et al., 2017; Lou et al., 2019). The previous studies revealed the key regulatory effect of ncRNAs in HCMV infections. Meanwhile, these findings also provided novel targets for HCMV treatment. For example, miR-221 alleviated CMV-induced tissue injury by promoting IFN- I and ISGs production (Yan et al., 2019). Furthermore, an inhibitor for miR-UL36-5p remarkedly reduced apoptosis mediated by miR-UL36-5p (Guo et al., 2015). Thus, ncRNAs could be developed as targets for anti-HCMV treatment. It has been proved that targeted ncRNA can effectively treat HCMV related disease in cell and animal models, but none clinical trials are reported so far. We sees a promising future for treatment HCMV related diseases by targeting ncRNAs in facilitating the translation of basic science to the clinical setting. However, how to specifically and efficiently regulate the targeted ncRNAs for HCMV therapy still need further studies.

## Author Contributions

ZY and BW: project administration and funding acquisition. ZY, JW, XZ, SJ, and WS: writing—original draft preparation. ZY, FN, and BW: writing—review and editing. All authors have read and agreed to the published version of the manuscript.

## Conflict of Interest

The authors declare that the research was conducted in the absence of any commercial or financial relationships that could be construed as a potential conflict of interest.

## Publisher’s Note

All claims expressed in this article are solely those of the authors and do not necessarily represent those of their affiliated organizations, or those of the publisher, the editors and the reviewers. Any product that may be evaluated in this article, or claim that may be made by its manufacturer, is not guaranteed or endorsed by the publisher.

## Abbreviations

HCMV: human cytomegalovirus
ncRNAs: non-coding RNAs
lncRNA: long non-coding RNA
circRNA: circular RNA
FOXO3: fork head box O3
IRF7: interferon regulatory factor 7
IER5: immediate early response gene 5
IE2: immediate early protein 2
VAC: virion assembly compartment
GATA-2: GATA binding protein 2
EGFR: epidermal growth factor receptor
GAB1: GRB2 associated binding protein 1
MEK: MAP kinse-ERK kinase
ERK: extracellular regulated MAP kinase
EGR1: early growth response 1
HUVECs: human umbilical vein endothelial cells
MOAP1: modulator of apoptosis 1
PHAP: putative HLA-DR-associated proteins
ERN1: endoplasmic reticulum to nucleus signaling 1
IEX-1: immediate early gene X-1
ANT3: adenine nucleotide translocator 3
HPCs: hematopoietic progenitor cells
QARS: glutaminyl-tRNA Synthetase
BCL2L11: BCL2 like 11
BRCC3: BRCA1/BRCA2-containing complex subunit 3
EID1: EP300 interacting inhibitor of differentiation 1
MAPRE2: microtubule associated protein RP/EB family member 2
TUSC3: tumor suppressor candidate 3
NSG: NOD scid gamma
GBM: glioblastoma multiforme
CSC: cancer stem cells
SIRT1: sirtuin 1
Enos: nitric oxide synthase
Sox2: SRY-box transcription factor 2
TLR2: toll like receptor 2
NAB1: NGFI-A binding protein
TGF- β: transforming growth factor beta
RANTES, Regulated on activation: normal T-cell expressed and secreted
CTLs: cytotoxic T lymphocytes
HLA-E: major histocompatibility complexclass IE
NF- κ B: nuclear factor kappa B
ISGs: IFN-stimulated genes
STAT1: signal transducer and activator of transcription 1
SOCS1: suppressor of cytokine signaling 1
IFN: type I interferon
NLRP3: NLR family pyrin domain containing 3
MCMV: murine cytomegalovirus
ET-1: endothelin 1
Ednra: endothelin A receptor
CHB: chronic hepatitis B
APOs: adverse pregnancy outcomes.
